# On the Psychological Effects of Certain Medicinal Agents

**Published:** 1848-04-01

**Authors:** 


### ON THE PSYCHOLOGICAL EFFECTS OF CERTAIN MEDICINAL AGENTS.

Every day's experience is adding something to our previously collected stores of information in psychology, yet how much more is to be gleaned, how many and how various are the sources from which we may yet anticipate valuable knowledge. Amongst the many phenomena which present themselves, none are more deserving investigation than the effects produced upon the manifestations of the mind by several drugs, which, although more immediately ranked as poisons, are administered fearlessly in medicine, because they have the power of diminishing some of the sufferings of the human body. Those who have watched the progress of disease, have often been surprised by the sudden alteration which they have perceived in the state of the senses of individuals, and have for some time been at a loss to account for it, when they have, from cautious investigation, arrived at the conclusion that it is attributable to a particular remedy employed; and upon consulting different authorities, they have found that a similar remark has been made by others, but no one has yet collected together the evidence that exists. This, indeed, is scattered amongst the periodical publications, and the works of writers on medical jurisprudence and toxicology, where the facts are simply stated, without any attempt to analyse or discuss them. Singular, however, are the numerous instances where the senses convey to the brain impressions inconsistent with those that are presented under ordinary circumstances, when the affections, emotions, feelings, and passions, which have before flowed on in an even current, have been violently disturbed and excited; when the faculties, the imagination, the memory, have been called into unwonted activity, or have been apparently in so exhausted a state as to have become incapable of being roused into action, or even altogether obliterated. Occasionally there has been such an unusual exhibition of singularities, as to excite an apprehension lest the whole of the mind should become involved in the derangement, sink into idiotic apathy, or be subject to intermittent paroxysms.

In perusing the writings that are handed down to us as the works of the most remarkable men of Greece and of Rome, we cannot fail to be struck with the allusions that are frequently made to the knowledge which existed of the power of drugs over the intellectual faculties. We perceive that they were fully acquainted with the means which modern science has only so recently furnished us with, of acting both upon body and mind, so as to produce either rapid or slow action. We are daily learning that many of those circumstances which were ascribed to the credulity, or even to the imagination of the historian, who carelessly collected the idle gossip or marvels of the age in which he lived, are within the pale of truth, and have been proved by the discoveries which have been made by the chemist. Neither the language of Plutarch, when speaking of the drugs that were administered to the younger Aratus for the purpose of depriving him of reason, "so that he took up inclinations that were shocking and monstrous," nor that of Tacitus, in describing the means taken "to disorder the brain of Claudius, and not to kill him," is inconsistent with our present state of knowledge of the agents which the hand of art has educed from the stores of the vegetable world. Many, certainly, are the difficulties which surround an inquiry into the peculiar influence of drugs upon the mind. If scientific men were altogether satisfied upon the constitution of that wondrous gift which elevates man above all the rest of created beings, we should better be enabled to enter upon the discussion; but whether its conditions and operations spring from one unvarying source, which governs and controls them, or whether it is an aggregate of powers and of faculties, each independent of the other, are questions which remain yet to be settled. It is, however, to be hoped that a collection of facts and of observations may yet be made, which will furnish us with more ample ground for a theory than we yet possess.

It is the class of remedies termed narcotics, that usually furnish drugs which act upon the cerebral functions. Their first stage is that of excitement, during which there is an increased energy of the faculties, variously exhibited, according to the nature of the medicine, the dose, and the peculiar idiosyncrasy of the individual; the second stage is that of reaction; the ultimate one, that of collapse; for the law seems universal that wherever there has been an increased energy of action, there shall also be an intensity of repose, during which it would appear that the exhaustion is relieved, and power imparted. There are also drugs which act upon the mind secondarily, their action being primarily upon the corporeal functions; they may depress the mind, but it is because they diminish the powers of the organs which give strength and nutrition to the system.

In some individuals a small dose of calomel will produce a state of morbid sensibility, which will be displayed during the whole time the system is under its action. An hysteric feeling, the shedding of tears, the yawning, the sensation of cold that accompanies this state, are singularly distressing; but the cheerfulness and accustomed serenity are restored the moment the action of the medicine has ceased. In such cases, the mucous membranes are generally highly susceptible, and catarrh and bronchitis are often the result, whilst the glandular system is also deranged. Iodine, after it has been persevered in for some length of time, acts most unequivocally upon the brain and nervous system. During a course of any of its compounds, many persons become depressed, unhappy, and doubtless where there is a predisposition to mental derangement, it has been called into action, and many are the cases which illustrate the power of this substance. Still, however, we are led to the conviction that the primary effect has been upon the functions of the different corporeal organs, and that these organs have been gradually so acted upon, as to be incapable of furnishing the secretions for which they were destined. It is the glandular system which they strikingly influence, producing absorption of the glands. In many instances, the mammæ in females, and the testes in men, have almost disappeared; nor have they ever again resumed their former state, nor has any lapse of time caused a deposition of tissue similar to that of which they were originally composed. On the other hand, the preparations of iron will impart a vigour and elasticity to the mind, excite cheerfulness, and be the cause of an improved state of feeling. Cinchona bark, cascarilla, and, to some extent, sarsaparilla, will likewise give a tone and temper to the mind; phosphorus has been much lauded by the Germans. But all these act by giving to the body energy and activity. A system of dietetics, no doubt, materially influences the mind. We have had strange tales recorded of our eminent painter, Fuseli, who ascribed his wild and unearthly imaginings to the raw beefsteaks on which he supped; when full of horrors, he seized his pencil, and gave his graphic illustrations. That many singular effects have thus been produced, and that diet exercises a strong influence upon the character and the mind, the history of nations teaches us. The Hindoo, who feeds upon rice and upon vegetables, is a meek, quiet being, governed by any nation that chooses to rule over him, whilst the savage disposition of the eater of raw flesh is known to every ethnologist. But it is not upon the secondary influence on the brain that the psychologist seeks to be informed; it is of that marvellous power which experience has taught us that opium, cannabis sativa, atropa belladonna, conium, and datura stramonium immediately produce, that accurate information is sought.

When the first travellers to the East recounted what they had seen amongst the opium eaters, they were suspected of exaggeration; time and experience have proved the truth of their statements, yet but little light has been thrown upon the points which most interest the physician and the psychologist, but from all that can be collected, it would appear that it is the imagination that is most generally acted upon, independent of that peculiar torpor which is accompanied by sensations of gratification, and absence of all communication with the external world. The senses convey no false impressions to the brain; all that is seen, heard, or felt is faithfully delineated, but the imagination clothes each object in its own fanciful garb. It exaggerates, it multiplies, it colours, it gives fantastic shapes; there is a new condition arising out of ordinary perception, and the reason, abandoning itself to the imagination, does not resist the delight of indulging in visions. If the eyes are closed, and nothing presented to excite the external senses, a whole train of vivid dreams are presented. A theatre is lighted up in the brain; graceful dancers perform the most captivating evolutions; music of an unearthly character floats along; poesy, whose harmonious numbers and whose exciting themes are far beyond the power of the human mind, is unceasingly poured forth. Memory is, however, generally asleep: all the passions, affections, emotions have lost their sway. It is all an exquisite indolence, during which dreams spontaneously arise, brilliant, beautiful, and exhilarating. There is order, harmony, tranquillity. If a single object has been vividly impressed upon the eye, it is multiplied a thousand times by the imagination. Many singular instances are narrated in the confessions of the Opium Eater of this effect. Vast processions passed across him in his reveries in mournful pomp. The species of suffering from the endless repetition of the Malay whom he had seen once, is accurately described. The reaction is, of course, frightful. The efforts of Nature to restore her lost equilibrium lead to a state which renders life loathsome, and the accustomed opiate becomes the only resource. The alterations that eventually occur in the mind are states of melancholy and of gloom, a tendency to suicidal despondency. But, singularly enough, the body exhibits the effects much more strikingly; the eye loses all its lustre, the complexion its natural hue; deformities of the person arise; wry necks, elevation of one shoulder above the other, contortions of the limbs, are its frequent consequences. Those who have been accustomed to opium, in consequence of its administration as a medicine, require doses which at length produce first the exhilaration, and, gradually, the morbid melancholy, which are amongst the marked characteristics of this drug. In lunatic asylums are occasionally found cases of deplorable melancholy, which have been produced by opium eating. They are said to be frequent in Turkey, but, as yet, we have not gleaned from statistic reports information accurate enough to be thoroughly depended upon.

Strenuous efforts have been made, both in England and in France, to introduce the cannabis Indica, or Indian hemp, into practice. Under the auspices of some of our practitioners in British India, who extolled it in the highest terms, it was administered as a narcotic and as an anodyne, but the substance was found far inferior to the drugs already employed to produce sleep and to alleviate pain; it has, therefore, rapidly fallen into disuse amongst us, and we have but few well-attested narratives of its powers. In France, however, it has been more generally tried, and the extraordinary circumstances which have followed upon its employment have called forth numerous testimonies. To Dr. Moreau, of Tours, however, the profession is deeply indebted, not only for a very able discourse upon hallucinations, but for the result of his own experience. He has related the circumstances which occurred to himself and to some of those who accompanied him on a visit to Egypt, in the year 1837: the high character that he bears, his official position, are guarantees of the fidelity of his narratives, and compel the most incredulous to give attention to the details, more especially since they are daily confirmed by the experience of those who have been administering in their practice this singular substance. It must likewise be borne in mind, that as early as the days of Prosper Alpinus, the marvellous effects of these "drowsy syrups of the East" have been in the mouths of the learned and the unlearned, and that our modern travellers, amongst them Lane, who have looked at the subject as a mere matter of curiosity, have related instances which appear almost incredible. In Asia Minor, an extract from the cannabis Indica, under the name of hachich, has been from time immemorial swallowed with the greatest avidity, as the means of procuring the most ecstatic delight, and affording a gratification even of a higher character than that which is known there to follow upon the use of opium. A moderate dose seems only to influence the moral faculties, giving to the intellectual powers greater vivacity and momentary vigour; whilst a large dose, as it were, awakens a new sensibility, and calls into action dormant capabilities of enjoyment. Not only is the imagination excited, but an intensity of energy pervades all the passions and affections of the mind; not only does memory recur to the past with facility, but delusions are incorporated with it,—for with whatever accuracy the facts may be remembered, they are painted with glowing colours, and made sources of pleasure. The senses become instruments also of deception: the eye and the ear are not only alive to every impression, but they delude the reason and disturb the brain by the delusions to which they become subject; gaiety or a soothing melancholy may be produced, as pleasant or as disagreeable sights or sounds are presented. So much alive to this effect of external objects upon the perceptive powers are the swallowers of the hachich, that they generally retire into the depths of the harem, where the almas, or females educated for the purpose, add, by the charms of music and the dance, to the false perceptions which the disordered condition of the brain gives rise to. Insensibly the reason and the volition are entirely overcome, and yield themselves up to the fantastic train of imagery which affords such ecstatic delight. At the commencement of the inebriation, there is the most perfect consciousness of the state of the disordered faculties: there exists the power of analysing the sensations, but the mind seems unwilling to resume its guiding and controlling power; it is conscious that all is but a dream, still it seems to delight in the feeling of perfect abandonment to false enjoyment; it will not attempt to awaken from the reverie, but rather to indulge in it to the utmost extent of which it is capable. There seems an ideal existence, but it is too pleasurable to shake off; it penetrates into the inmost recesses of the body,—it envelops it,—the dreams and phantoms of the imagination appear part of the living being; still there is the internal conviction that the real world is abandoned for a fictitious and imaginative existence—it is too delightful to resist. To the extreme rapidity with which ideas, sensations, desires, rush across the brain may be attributed the singular retardation of time, which appears to be lengthened out to eternity.

Some singular illustrations of this peculiar state are given by Dr. Moreau. On one occasion he took a dose of the hachich previously to his going to the opera, and he fancied that he was upwards of three hours finding his way through the passage leading to it. Whilst the indefinable sensation of happiness so completely takes possession of the individual, and the joy and exhilaration are felt almost too great to be borne, the mind seems totally at a loss to account for it, to explain from what particular source it springs. There is a positive sensation of universal contentment, but it is vain to attempt to explain the nature of the enjoyment: if the inquiry be made, what is the peculiar emotion? the general answer is, that it is inexplicable, that the delicious sense of something unusual pervades every fibre, but any attempt to analyse or to describe it is vain. After some length of time, it would appear that the system is no longer capable of further happiness; the sensibility seems thoroughly exhausted,—a gentle sense of lassitude, physical and moral, gradually succeeds,—an apathy, a carelessness, an absolute calm, from which no external object can arouse the torpid frame, is the marked characteristic of this stage. The most alarming or afflicting intelligence is listened to without exciting any emotion. The mind is thoroughly absorbed: in this quiescent state, the perception seems blunted, the senses scarcely convey any impression to the brain; a reaction has taken place, yet the collapse is unattended with any disagreeable feeling; the energies are all prostrate, still there are none of those depressing symptoms which attend the last stages of ordinary inebriation. All that is described is an ineffable tranquillity of soul, during which it is perfectly inaccessible to sorrow or to pain—this imaginary beatitude, which those only who have felt can only describe. It is not unfrequent for illusions and hallucinations to occur during the first stage, when the external senses have lost their power of communicating faithfully to the brain the impressions they receive. Dr. Auber, in his work on the plague, relates several instances of delusion occurring during his administration of the cannabis Indica for the relief of that fearful disease. An officer in the navy saw puppets dancing on the roof of his cabin; another believed that he was transformed into the piston of a steam-engine; a young artist imagined that his body was endowed with such elasticity, as to enable him to enter into a bottle, and remain there at his ease. Dr. Moreau, on one occasion, believed that he was melting away by the heat of the sun; at another, that his whole body was inflated like a balloon, that he was enabled to elevate himself and vanish in the air. The ideas that generally presented themselves to him of these illusions was, that objects wore the semblance of phantasmagoric figures, small at first, then gradually enlarging, then suddenly becoming enormous and vanishing. Sometimes these figures were subjects of alarm to him: a little hideous dwarf, clothed in the dress of the thirteenth century, haunted him for some time; aware of the illusion, he entreated that the objects which kept up the illusion should be removed,—these were a hat and a coat upon a neighbouring table. An old servant of seventy-one was, upon another occasion, represented by his eye to the brain as a young lady, adorned with all the grace of beauty, and his white hair and wrinkles transformed into irresistible attractions. A friend who presented him with a glass of lemonade was painted to his disordered imagination as a furnace of hot charcoal. Sometimes the happiness was interrupted by delusions that affrighted him: thus, having indulged himself with his accustomed dose, he was suddenly seized with a panophobia; every object awoke his terror and alarm, which neither the conviction of his own mind nor the soothing explanations of his friends could diminish, and he was for a considerable length of time under the most fearful impressions.

These are the immediate effects of this toxicological substance, but there are others of a much more singular character, and which deserve to be studied by the psychologist; they are those which have called forth the observations of Lane and of other travellers, and have become the objects of a more intense curiosity, because they are not to be observed under the influence of any known substance, whether dietetic or medicinal: they are also so marvellous as to have raised doubts in the minds of the incredulous, which, however, have been most generally set at rest by the undoubted occurrence of the phenomena. Those who have witnessed the fearful symptoms betrayed during the paroxysms of delirium tremens,—who have heard sufferers declare that they saw before them genii, fairies, devils,—know how much the senses may then become the sources of delusion, and they can judge what the disordered state of intellect leads to. But not only during the immediate access of the disease caused by intoxication, but at other times, will the drunkard, confirmed in his habits, have these false perceptions, if he has at any time laboured under the characteristic delirium. When the brain has once been subject to be disordered by the use of the hachich, it becomes liable to delusions and hallucinations, different from those produced by anything but alcohol, after delirium tremens, and the mind believes that it sees visions, and beings with whom it can converse. This phenomenon is gradually developed, and a chronic disorder of the faculties of the brain may be said to exist; illusions are taken for realities, they retain complete possession of the mind, which upon all other points seems to be in its state of health; credulity, a vivid faith, and strong prejudice confirm it in its belief, which neither argument nor ridicule can shake. The Arabs, especially those of Egypt, are exceedingly superstitious, and there is scarcely an individual, even among the best informed, who does not believe in the existence of genii. These were, according to their belief, created before Adam; they are an intermediate class between angels and men; they are formed of an igneous substance, and can at their pleasure assume the form of men, of animals, or monsters, or can render themselves invisible. They are subject to death, but live many ages. Scarcely an eater of the hachich whose disordered fancy does not lead him to believe that he has either seen or is in immediate communication with one of these ideal beings. Mr. Lane had a cook, usually very gay, who was addicted to the hachich. One evening he found him on the staircase, with a frightened air, addressing an imaginary being, whom he most politely invited into the kitchen, and apparently repeated his request to no effect; on Mr. Lane expressing his astonishment, and asking him whom he was speaking with, he said that it was to an effrie, or genius, in the Turkish costume, who was smoking his pipe on the staircase, and had just come out of the well in the court. When Mr. Lane assured him that he saw no one, the cook observed that it might be so, and that was because he had a pure conscience. Dr. Moreau has given us many instances, from his own immediate followers, of genii seers amongst the hachich eaters. His dragoman, who had been attached in that capacity to Champollion, a man of superior sense, the captain of the vessel, and several of the sailors, had not only a firm belief, but had actually received several visits from genii, or effries, and neither argument nor ridicule could shake this conviction. The reis, or captain, had on two occasions seen a genius; he appeared to him under the form of a sheep. On returning one evening somewhat late to his house, the captain found a stray sheep, bleating with unusual noise; he took him home, sheared him for his long fleece, and was about to kill him, when suddenly the sheep rose up to the height of twenty feet, in the form of a black man, and in a voice of thunder, announced himself as a genius. One of the sailors, Mansour, a man who had made nearly twenty voyages with Europeans, recounted his interview with a genius under the guise of a young girl of eight or ten years; he met her in the evening, on the banks of the Nile, weeping deplorably because she had lost her way. Mansour, touched with compassion, took her home with him; in the morning he mounted her on an ass, to take her to her parents. On entering a grove of palms, he heard behind him some fearful sighs; on looking round to ascertain the cause, he saw, to his horror, that the little girl had dismounted, that her lower extremities had become of an enormous length, resembling two frightful serpents, which she trailed after her in the sand. Her arms became lengthened out, her face mounted up into the skies, black as charcoal; her immense mouth, armed with crocodiles' teeth, vomited forth flame: poor Mansour suddenly fell upon the earth, where, overcome with terror, he passed the night. In the morning he crawled home, and two months of illness attested the fact of cerebral disorder. Many such tales are recounted, and all told by the sufferers with the firmest belief and the most earnest conviction of their truth, each by his own delusion strengthening and confirming others. All the persons who thus had seen visions had already their brain in a state of disease, from the habit of eating the hachich, whilst those who do not indulge in the pernicious drug are free from such extraordinary hallucinations. Those who have abandoned the hachich, and have followed a hospital regimen, have been relieved, not only from the disordered state of sense which has led to their illusions, but they have doubted their own soundness of mind when they gave way to them. The fact that these hallucinations are entirely independent of any affection of the brain at the moment they occur, and that the individual is, under every other circumstance, fitted for his usual avocations of life, renders this subject one of deep interest. Hallucinations may be only symptoms that the intellect has been previously disordered, but they frequently become the point from which insanity is developed; for, in ordinary mental illusions, the senses, the reason, and the volition, become in a short space of time impaired, the passions and affections, if unrestrained, soon aggravate the condition, and absolute insanity succeeds. Hence, in all such cases occurring in ordinary life, the necessity of watchfulness has been inculcated by the most judicious physicians.

In the more recent publication which Dr. Moreau has given to the medical world, he has, with great ingenuity and with much ability, attempted to show that a vast number of the affections of the mind may be studied and elucidated by comparing them with the effects produced by the hachich ; from the great opportunities which are afforded him at the Bicetre and at Ivry, there may be expected, as well as from his talents and his assiduity, a mass of information useful to society at large.

The cerebral disturbances which result from the datura stramonium are exceedingly striking, and have also called forth the observation of medical men, rather as a curious anomaly than as worthy more precise investigation. The earliest information of its effects that we possess rather led to the belief that idiocy followed upon its use, and that the loss of the perceptive powers, and consequent dementia, were its usual sequelæ. A species of delirium, which has lately been denominated by psychologists, a fantasia, takes place. The mind appears to be under the influence of a troubled dream, and the person is subject to bursts of uncontrollable laughter. The quaint language of Beverley, the historian of Jamaica, fully describes the effects of the thorn apple. Some soldiers, who were sent to quell the rebellion in the island, ate of it: "the effect was a very pleasant comedy, for they turned natural fools upon it, for several days. One would blow up a feather in the air, another would dart straws at it with much fury; another, stark naked, was sitting up in a corner grinning like a monkey, and making mouths at them; a fourth would fondly kiss and paw his companions, and sneer in their faces with a countenance more antic than a Dutch doll. In this frantic condition they were confined, lest in their folly they should destroy themselves. A thousand simple tricks they played, and after eleven days, returning to themselves again, not remembering anything that had occurred." When stramonium, in the form of extract, has been injudiciously administered, it has occasionally affected the perceptive senses, especially the sight. Imaginary objects are seen to play before the eyes, at which the individual strikes, as they seem to terrify him. Where the seeds have been taken, a similar effect occurs. A case is detailed by Fowler, of a child, who supposed that cats, dogs, rabbits, were running on the tops, sides, and middle of the room. When inhaled for the purpose of relieving the paroxysm of asthma, the smoke conveys a sense of gentle tranquillity; the muscles of the thorax, and those which have been laboriously called into motion to assist them, until spasmodic action has been produced, are at once rendered less irritable, and the fibre is relaxed, sleep is induced, but there is rarely any disturbance of the imagination. The belief expressed by Kæmpfer, that this substance is used by the Orientalists as a substitute for opium and the cannabis Indica, is not generally entertained; but Garzia dall Horto affirms that the natives of Malabar use it for the purpose of deadening the senses of any individual whom they intend to pillage; that under such circumstances all power of memory is lost, and that the only evidence given of the action of the stramonium, has been the uncontrollable laughter which bursts from the person thus treated. Faber has also alluded to this apparent merriment, telling us in language that need not be translated, "Decoctum daturæ maritis propinnat, quo fit ut apertis oculis, nequitium et turpitudinem proposiam inspicientes, mente tamen turbate, cachinno tantura seipsos deludunt, ac subsannatium morsus probant." As this is said of the Turkish ladies, usually shut up in harems, where there would be some difficulty to obtain the herb, and thus to use it, it may rank amongst the "incredibilia" which detract so much from medical literature. That there are frightful cases on record on the Continent, where wretches, under the form of men, have administered the decoction of stramonium to helpless girls, with a view of committing crime upon their persons, and have unexpectedly consigned their miserable victims to hopeless insanity, and to a train of cerebral symptoms of a most frightful character, we cannot but be aware: they are fortunately unknown in this country, and we will not drag them from the obscurity in which they are now enveloped.

The atropa belladonna is a plant of which we in reality know but little, notwithstanding it has kept villages in alarm, and has been the cause of many a country doctor in former days rising into provincial reputation. Its berries have been the dread and bane of mothers, who have at the autumnal season given many a warning, soon thoroughly diregarded, to their children, as they sallied into the woods, not to gather them, however tempting they may look. Of the circumstances which attended the introduction into practice of the different parts of the plant, we must leave the writers of materia medica to speak; but that it produced hallucinations, we may judge from the derivation, if, at least, the etymologists are correct, who tell us that it was called belladonna, because those to whom it was administered saw beautiful females before them. That it produces illusions of a singular character, there can be little doubt, nor have we any reason for denying that cases of decided impulsive insanity have been produced by it when given in repeated doses. Some of the most frightful cases of suicidal impulse, and of destruction, have been declared to have had their development from its use, where previous predisposition to insanity had existed. It has been asserted, that where tic doloureux has been suddenly stopped by belladonna, this form of madness has been called into action. The French physicians have attributed the sudden outbreak of insanity in a distinguished individual, who recently attempted, or rather threatened the murder of his children, to a similar cause; certain it is that there were many reasons which induced this belief. But the effect of belladonna upon the cerebral mass is of a most extraordinary character, and unlike any of those which are usually found to be attendant on the other narcotics. A moderate dose disturbs the sentient powers, yet all objects appear to them in their natural and ordinary form; but they seem irritated by their presence, and they call into action the muscular powers, which, seeming to obey the will, attempt to grasp and seize them, though the reason and judgment would inform them that these objects were at too great a distance. One of the marvellous effects of continued doses, is the production of a singular psychological phenomenon; a delirium supervenes, unaccompanied with any fantasia or imaginary illusion, whose marked characteristic is somnambulism. An individual, who has tak[illegible] it in several doses, seems to be perfectly alive to surrounding objects; his senses conveying faithfully to the brain the impressions that they receive; he goes through his usual avocations without exhibiting any unwonted feeling, yet is he quite unconscious of his existence, and performs mechanically all that he is accustomed to do; answers questions correctly without knowing from whom, or from whence they proceed; looks at objects vacantly; moves his lips as if conversing, yet utters not a sound; there is no unusual state of the respiratory organs, no alteration of the pulse, nothing that can bespeak excitement. When this state of somnambulism passes away, the individual has not the slightest recollection of what has occurred to him; he reverts to that which immediately preceded the attack, nor can any allusion to his apparent reverie induce him to believe that he has excited any attention. The case of the tailor, who remained on his shopboard for fifteen hours, performing his usual avocations, sewing with great apparent earnestness, using all the gestures which his business requires, moving his lips as if speaking, yet the whole of the time perfectly insensible, has been frequently quoted. It was produced by an injection of belladonna; and it is a fact now well ascertained, that drugs will act upon the brain, exhibit their peculiar odour in the breath much more rapidly, and with much greater certainty, if they are injected into the rectum; this ought to be borne in mind by those who are called upon to administer narcotics in the form of enema.

A curious fact occurred at the time that Hufeland's letter was published in the "Lancet," on the subject of the prevention of scarlatina, when it appears under the form of an epidemic, by the administration of belladonna in repeated doses. A family, consisting of eleven persons, had been strongly recommended by the physician who had been called upon to attend a little girl about three years of age, who had sickened with scarlatina, to try the proposed prophylactic. No sooner was the redness of the skin perceptible, and soreness of the throat complained of, than to each person was duly given ten drops of a solution, consisting of five grains of belladonna in two ounces of water, twice in each day; five of these persons were domestics; almost all of them, on the fourth day, became under the influence of the drug, two or three of them very slightly, simply complaining of having the vision disturbed by objects which they in vain attempted to remove, for they were fully persuaded that they existed; two had singular fits of laughter, which nothing could control; all of them complained of being in an unusual state; the servants were all of them able to go through their work, but all seemed to act mechanically, each independent of the other; of this the most ludicrous example was in the course of the fourth evening: a carriage arrived at the door, and the street-bell was rung with considerable violence; they all immediately left their business, quietly walked up-stairs, as if they had not the slightest idea that they were all upon the same errand; they went to the door, two of them, however, only opened it,—one of these walked away without waiting to know what was the reason of the ringing, and the other seemed not disposed to trouble himself with anything beyond the opening and shutting the door. On the discontinuance of the medicine, they all soon returned to their usual state, and two of them had scarlatina, though the disease appeared under a mild form. Many instances of somnambulism have been attributed to the use of the belladonna, and certainly, what is known of its operation seems to bear out the idea that it acts upon the cerebral system. These facts are worthy the deepest attention of the psychologist. The employment of belladonna for the cure of insanity has been strongly recommended by the school of Hahnneman, upon the grounds of its power of producing the worst forms of mania when taken in inordinate doses. That the more violent passions have been evinced by those who have taken it in repeated doses, there can be but little doubt; and even those whose temper and disposition have always held the calm current of their way, undisturbed by any of those extravagant emotions to which some of the race of mortals are subject, have been known to be exceedingly irascible, and have displayed the most extravagant marks of passion. These ebullitions have not been temporary, but occasionally permanent; and where a predisposition to mania has existed, have terminated in an alarming crisis. Hyoscyamus, so generally substituted for opium, where there are indications which point out the impropriety of administering it, such as a brown tongue, heated breath, confined bowels, arterial determination, or venous retardation in the cerebral vessels, is a remedy not long to be persevered in; for it, like belladonna, produces anger, and amongst other singular phenomena, jealousy of the most malignant character is developed. Not only that passion, which is generally associated with love and the gentler emotions of the heart, but a furious hatred, envy, and dislike, are exhibited towards those persons with whom in the ordinary course of life patients are thrown, whilst under its influence. The great attention paid by Hahnneman to the effect of drugs, however much we may be disinclined to admit the benefit of his infinitesimal doses, makes him a high authority,—indeed, his work on Arsenic, written before the theories got possession of his mind, is a standard book, more valuable than any we possess on the poison.

He observes, that "a man, who became deranged through jealousy, was for a long time tormented by a physician with remedies that produced no effect upon him, when, under the name of a soporific, he one day administered hyoscyamus, which cured him speedily. Had he known that this plant excites jealousy and madness in persons who are in health, and had he been acquainted with the homœopathic law, the sole natural basis of therapeutics, he would have been able to administer hyoscyamus from the commencement with perfect confidence, and thus have avoided fatiguing the patient with remedies which could be of no service." In the German Ephemerides, in the Dictionnaire de Médecine, are related several well authenticated cases of the power of the odour of the leaves and of the fumes of the seeds of henbane over the more intense passions. A disposition to quarrel and to fight has been decidedly produced. One case is well described—viz., that of a couple who had married from affection—had lived upon terms of the most perfect mutual regard—indeed, they had been noticed for the warmth and strength of their attachment; but suddenly, to the surprise of the surrounding neighbours, their harmony was not only interrupted, but they became bitter antagonists, fighting and beating each other most unmercifully. What seemed most surprising was, that in one particular room appeared to spring their most determined quarrels, and that they soon subsided elsewhere. This mystery was at length explained, and their days of happiness restored, by the discovery, that, to the effects of a considerable quantity of hyoscyamus stored up for drying, their miseries were owing; and on the removal of this, the source of their feuds appeared to vanish. Camphor has many symptoms peculiar to itself. The common camphor pulp, in a medium dose, gently elevates the spirits, produces cheerfulness, and causes a flow of words; but a little beyond the usual quantity, and there will be indistinctness of ideas, incoherence of language, an indescribable uneasiness, shedding of tears, a sensation of fear and dread; then the body feels lighter than usual; an idea exists that flying will not only be easy, but a source of pleasure. In the case of Dr. Edwards, of Paris, mentioned by Orfila, a camphor injection produced phenomena of this kind, and on going down stairs to call for assistance, he was surprised to find his body appearing so light, that he seemed to skim along the surface of the floor without touching it. In what way digitalis acts upon the brain, psychologists have examined without arriving at any satisfactory conclusion. That it diminishes the irritability of the muscular fibre of the heart is universally acknowledged, and its sedative power, after repeated doses, is exhibited by the repeated swoonings, during which, in several instances, patients have died. That the brain loses its powers owing to the general enfeebling of the system, is well known; but that which is at present inexplicable, is the singular loss of memory that has been noticed. An individual under its influence loses all recollection of the events of yesterday, in the first instance; he then forgets the names of things and persons; but to what extent the impairing of this faculty may be carried, we have no opportunities of judging. Those who have been relieved by digitalis from dropsy are generally persons debilitated and worn out by disease, and especially those who have been subjected to mercurial treatment, so that it may be inferred almost generally, that the organs are more or less in an unsound state, and hence their functions are feebly performed, and amongst those that are thus affected must be the brain. A copious inhalation of the fumes of tobacco leaves has produced a singular species of derangement, of which Mr. Howison, in his voyage, gives us an example in his own person,—frightful dreams, a species of trance, during which sounds were not heard through the sense of hearing, but by a vibration throughout his body, are amongst the symptoms he describes. The dreadful effects from its external application in skin diseases upon the brain and nervous system have often been narrated, but the general torpor which they undergo is the result of oppression from a narcotic aerial fluid circulating with the blood, so that its action is not thoroughly known.

---

